# Abundance and Size of Gulf Shrimp in Louisiana's Coastal Estuaries following the Deepwater Horizon Oil Spill

**DOI:** 10.1371/journal.pone.0108884

**Published:** 2014-10-01

**Authors:** Joris L. van der Ham, Kim de Mutsert

**Affiliations:** Department of Environmental Science and Policy, George Mason University, Fairfax, Virginia, United States of America; James Cook University, Australia

## Abstract

The *Deepwater Horizon* oil spill impacted Louisiana's coastal estuaries physically, chemically, and biologically. To better understand the ecological consequences of this oil spill on Louisiana estuaries, we compared the abundance and size of two Gulf shrimp species (*Farfantepeneus aztecus* and *Litopeneus setiferus*) in heavily affected and relatively unaffected estuaries, before and after the oil spill. Two datasets were used to conduct this study: data on shrimp abundance and size before the spill were available from Louisiana Department of Wildlife and Fisheries (LDWF). Data on shrimp abundance and size from after the spill were independently collected by the authors and by LDWF. Using a Before-After-Control-Impact with Paired sampling (BACIP) design with monthly samples of two selected basins, we found brown shrimp to become more abundant and the mean size of white shrimp to become smaller. Using a BACIP with data on successive shrimp year-classes of multiple basins, we found both species to become more abundant in basins that were affected by the spill, while mean shrimp size either not change after the spill, or increased in both affected and unaffected basins. We conclude that following the oil spill abundances of both species increased within affected estuaries, whereas mean size may have been unaffected. We propose two factors that may have caused these results: 1) exposure to polycyclic aromatic hydrocarbons (PAHs) may have reduced the growth rate of shrimp, resulting in a delayed movement of shrimp to offshore habitats, and an increase of within-estuary shrimp abundance, and 2) fishing closures established immediately after the spill, may have resulted in decreased fishing effort and an increase in shrimp abundance. This study accentuates the complexities in determining ecological effects of oil spills, and the need of studies on the organismal level to reveal cause-and-effect relationships of such events.

## Introduction

As result of the *Deepwater Horizon* oil spill in April 2010 (referred to as the spill hereinafter), a large amount of spilled oil was introduced in the coastal estuaries of eastern Louisiana. Oil washed up on hundreds of kilometers of estuary shoreline and aggregated in bottom sediments of shallow coastal estuaries [1], [2]. As the oil entered coastal estuaries it was in various stages of chemical and physical degradation [3]. The oil nevertheless affected microbial communities [1], [4], marsh vegetation [2], [5], (semi-) terrestrial arthropod communities [6], and nekton [7].

Louisiana's coastal estuaries function as nursery habitat for numerous species, including brown shrimp (*Farfantepenaeus aztecus*) and white shrimp (*Litopenaeus setiferus*). The shrimp larvae drift into estuaries through tidal action as postlarvae, and stay in estuaries throughout their juvenile stages until they move offshore to mature and spawn [8], [9]. The two species enter estuaries as postlarvae during different periods of the year: brown shrimp typically from February to April, white shrimp from May to November. Shrimp of both species initiate their migration off-shore when they reach a threshold size [10]. The asynchronous lifecycles of both species causes a single calendar year to represent one brown shrimp year-class and two white shrimp year-classes, one that entered the estuary late in the previous year and another that entered starting in May. During and after the spill all life-stages of the shrimp life cycle could potentially have been exposed to contaminants. These contaminants include PAHs, which are natural components of crude oil. Laboratory studies have shown that PAHs reduce growth rates of decapod crustaceans by increasing the intermolt period and decreasing the growth increment per molt [11]–[14]. Exposure to PAHs can also result in increased mortality rates in crustaceans [15], [16] and affect various other physiological and behavioral aspects of crustacean biology [17]–[19]. Such contamination may have been the cause for the immediate decrease in crab burrows following the spill as described by McCall and Pennings [6]. While Gulf shrimp are consequently likely to suffer negative effects of exposure to oil, the short generation time of Gulf shrimp results in a fast population turnover, which generally results in a quick recovery on a population scale. For this and others reasons, shrimp populations have been predicted not to suffer dramatic effects as result of the spill [20].

In this study we investigated effects of the spill on the abundance and size of brown and white shrimp in Louisiana's coastal estuaries. We compared abundance and size of shrimp in estuaries that were heavily impacted by the spill with minimally impacted estuaries, both before and after the spill. We used two independent sources for the post-spill data, and included multiple successive year-classes for both species.

## Methods

### Research area

**Figure 1:**
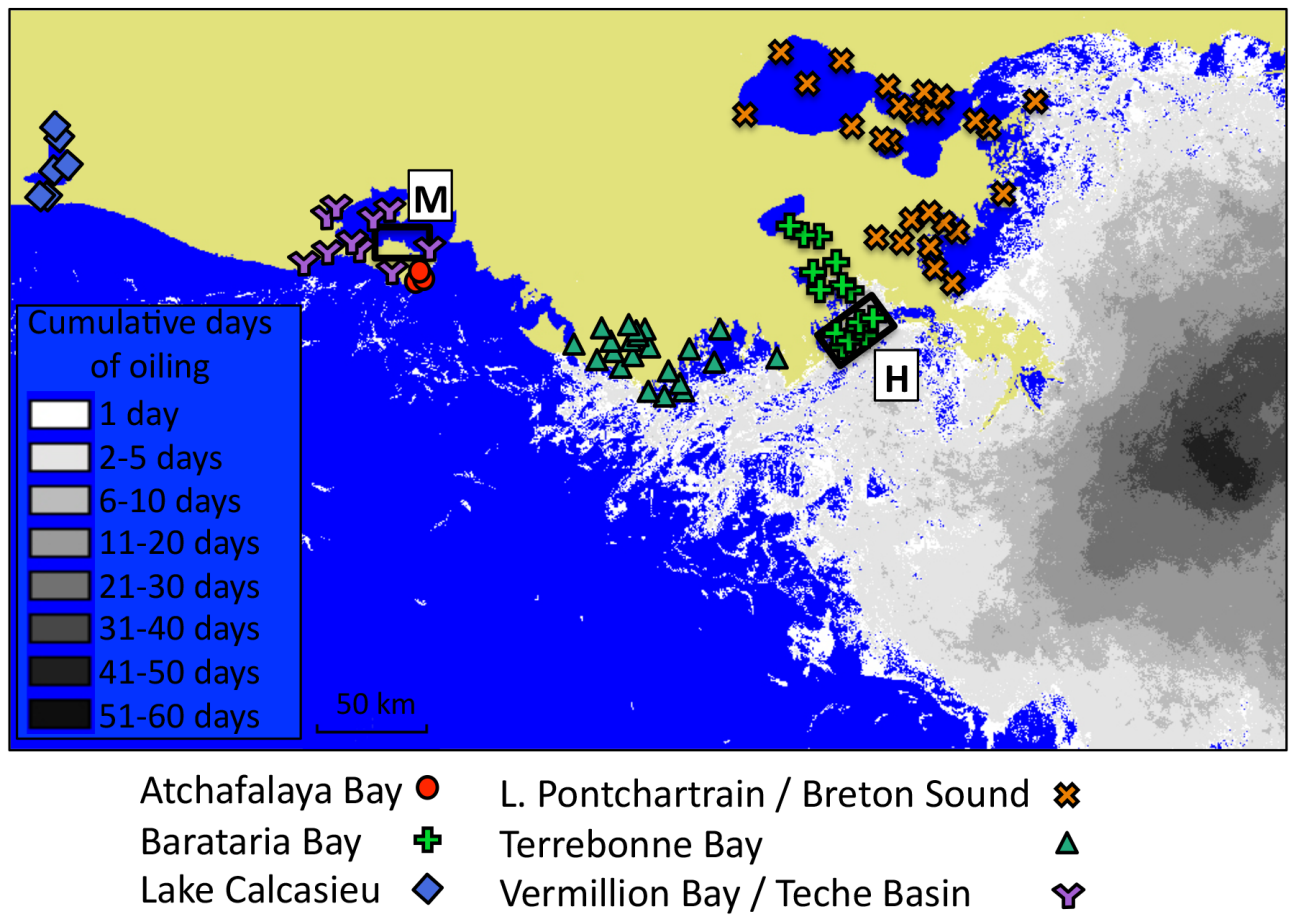
Louisiana coast showing the occurrence of spilled oil. As reported by NOAA's Environmental Response Management Application [26]. The shade of grey indicated the cumulative oiling (cumulative TCNNA SAR oiling). The FIMP sampling locations used in this study are indicated on the map. The bounded rectangles indicate areas including sites sampled by the authors. Minimally impacted sites (M), and heavily impacted sites (H).

We used two sets of research sites for two different analyses. Two minimally impacted sites in Vermillion Bay and two heavily impacted sites in Barataria Bay (Figure 1) were selected for a small-scale analysis. Vermillion Bay received little to no oil after the spill and the shoreline at these sites in this bay were pristine without any trace of washed up oil. Barataria Bay received relatively large amounts of oil and the shorelines near the selected sites in this bay were heavily oiled. A large percentage of the shoreline vegetation at these sites disappeared in the months following the spill, and all shrimp were collected in close proximity to the marsh shoreline. All four selected sited were long-term monitoring sites of the Fisheries Independent Monitoring Program (FIMP) conducted by the Marine Fisheries Division of the Louisiana Department of Wildlife and Fisheries. For the large-scale analysis, we included data collected at a varying number of sites in the following basins: Atchafalaya Bay, Barataria Bay, Lake Calcasieu, Lake Pontchartrain/Breton Sound, Terrebonne Bay, and Vermillion Bay/Teche Basin (Figure 1).

### Field methods

The small-scale analysis sites were sampled approximately monthly by the FIMP from 1987 to May 2010, and by the authors from June to October 2010, and from March to May 2011. All collections were made under scientific collecting permits issued by the state of Louisiana, Department of Wildlife and Fisheries. Sites included in the large-scale analysis were also sampled approximately monthly by the FIMP for a variable number of years since 1987 until 2012 (Table 1). All shrimp were collected monthly using a 4.9 m otter trawl, in duplicate, with 5 min trawls at 5 km/h. Abundance was measured as number of individuals per trawl, and size was measured as mean individual total length per trawl.

**Table 1:** Number of FIMP sampling stations per basin and year-class.

Species	Year-class	Months analyzed	Atchafalaya Bay	Barataria Bay	Lake Calcasieu	L. Pontchartrain/Breton Sound	Terrebonne Bay	Vermillion Bay/Teche Basin
Brown shrimp	2010	May ‘10–Dec ‘10	3 (408; 13)	8 (994; 47)	6 (802; 30)	16 (1283; 65)	22 (1570; 61)	8 (1128; 46)
	2011	Jan ‘11–Dec ‘11	3 (474; 24)	14 (1391; 105)	6 (1060; 39)	7 (1071; 23)	15 (2313; 106)	8 (1349; 65)
	2012	Jan ‘12–Jun ‘12	3 (174; 7)	8 (706; 38)	6 (520; 21)	13 (729; 27)	15 (1175; 76)	8 (557; 34)
White shrimp	2011	May ‘10–Apr ‘11	3 (677; 34)	14 (1406; 89)	6 (1397; 53)	18 (1721; 120)	15 (2671; 135)	8 (1827; 90)
	2012	May ‘11–Apr ‘12	3 (677; 36)	13 (1405; 129)	6 (1397; 58)	9 (1357; 28)	15 (2671; 149)	8 (1827; 96)

In parentheses the number of monthly pre-spill and post-spill observations since 1987, respectively.

### Analyses

In the small-scale analysis we compared monthly shrimp abundance and size of two heavily impacted sites with two minimally impacted sites, both before and after the spill. We employed a Before-After-Control-Impact design with Paired sampling (BACIP) design to analyze the data [21]. Per month, we compared the difference in each parameter (size and abundance of shrimp) between heavily impacted and minimally impacted sites before the spill (Δ_before_), and the difference in these parameters between heavily impacted and minimally impacted sites after the spill (Δ_after_). The average Δ_before_ is an estimate of the average difference between the two types of sites, which provides an estimate of the expected Δ_after_ in the absence of an environmental impact (i.e. the null hypothesis; [22]). A significant difference between Δ_before_ and Δ_after_ (i.e. site*time) indicates an environmental impact. We used size and abundance data collected by the FIMP prior to the spill as ‘before’ data, and our own collections at the same sites following the spill as ‘after’ data.

**Figure 2:**
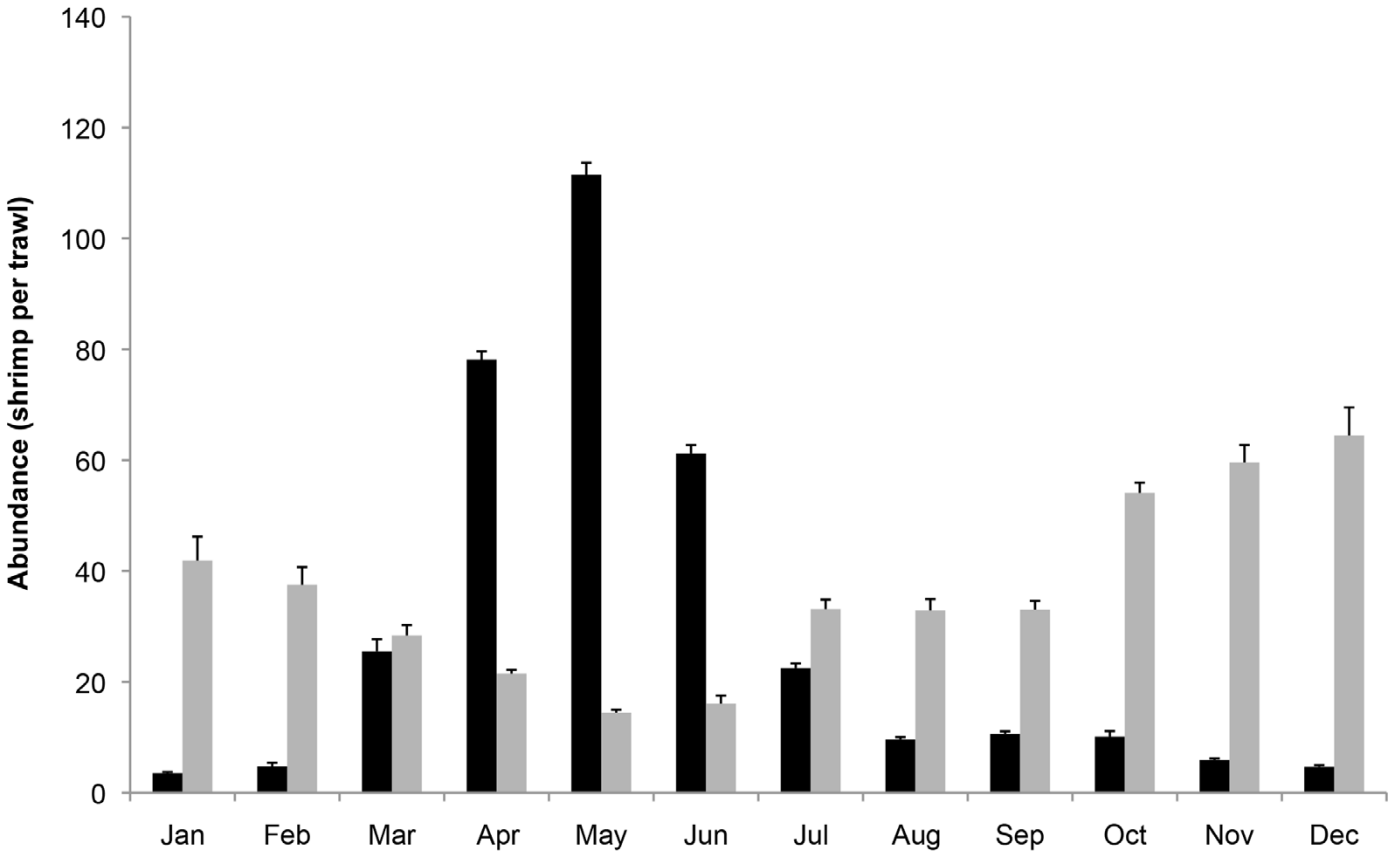
Mean within-estuary abundance of brown shrimp and white shrimp per month. Abundance (individuals per trawl) based on FIMP data of all selected sites since 1987 until the spill, black bars indicate brown shrimp, white bars indicate white shrimp. Using these data, one year-class of brown shrimp is measured from January to December, while one year-class of white shrimp is measured from May to April.

In the large-scale analysis we compared abundance and mean shrimp size of year-classes before the spill with those same metrics after the spill, per basin. This approach allows us to evaluate if small-scale patterns also occur on a larger temporal scale (months vs. year-classes) and spatial scale (two-basins vs. all Louisiana basins). This large-scale analysis was solely based on FIMP-data, which were collected in all basins at numerous sites along the Louisiana coast that fall on a gradient of heavily impacted to minimally impacted, which roughly corresponds with distance from the spill (Figure 1). Sites used in the small-scale analysis were excluded from the large-scale analysis, as well as data collected with methods other than a 4.9 m otter trawl. We determined year-classes for each species *a priori* using FIMP data of all selected sites since 1987 until the spill (Figure 2). We define year-classes as 12-month periods starting at a month of low abundance. Based on these empirical data, we define brown shrimp year-classes to start in January and end in December, and white shrimp year-classes to start in May and end in April. Due to the timing of the spill and the availability of the FIMP data, we included one complete year-class and two partial year-classes for brown shrimp, and two complete year-classes for white shrimp (Table 1) in the analysis.

In both analyses, Box-Cox transformations were performed where necessary to comply with normality and equal variance. Analyses of variance (split-plot model design with random sub-sampling) were used to determine significant effects, and least square mean (LSmean) contrast tests to estimate BACIP (small-scale analysis) and before-after contrast (large-scale analysis) [23]. All statistical analyses were preformed using JMP PRO v10.0 [24].

## Results

For the small-scale analysis 219,179 shrimp from 1,617 trawls were analyzed; for the large-scale analysis 2,178,859 shrimp from 30,185 trawls were analyzed. For the large-scale analysis nontransformed averages are provided in table 2. We found significant interactions in our BACIP design during some months of the small-scale analysis for both abundance and size of shrimp, indicating significant effects of the oil spill on shrimp. Specifically, in June and October 2010, brown shrimp were more abundant in the heavily impacted sites after the spill than before, whereas abundances in the minimally impacted sites did not significantly change (June, *p*<0.05, BACIP contrast (Box-Cox transformed)  = 86.8 (SE 40.7); October, *p*<0.05, BACIP contrast (Box-Cox transformed)  = 10.3 (SE 4.4)). During July and October 2010, and April, 2011 white shrimp were found to be smaller in the heavily impacted sites after the spill, whereas in the minimally impacted sites sizes were not significantly different (July, *p*<0.05, BACIP contrast  = 5.2 (SE 2.0); October, *p*<0.05, BACIP contrast  = 3.8 (SE 1.2); April, *p*<0.05, BACIP contrast  = 6.0 (SE 2.4)). During all other months shrimp abundance and size did not differ significantly during those same months prior to the spill.

**Table 2:** Average abundance (individuals per trawl) and size (mean size per trawl) for brown and white shrimp before the spill (1987–2010) and for year-classes following the spill.

			Abundance	Size
Atchafalaya Bay	Brown shrimp	Pre-spill	32.26 (2.30)	5.74 (0.08)
		2010	63.00 (34.62)	5.07 (0.62)
		2011	44.47 (15.00)	4.60 (0.25)
		2012	70.73 (14.55)	3.53 (0.53)
	White shrimp	Pre-spill	52.21 (3.96)	7.55 (0.18)
		2011	77.80 (16.73)	8.07 (0.56)
		2012	69.83 (13.41)	6.92 (0.49)
Barataria Bay	Brown shrimp	Pre-spill	39.64 (1.91)	7.64 (0.04)
		2010	55.35 (11.91)	7.27 (0.22)
		2011	140.82 (15.52)	5.62 (0.19)
		2012	116.43 (19.77)	5.86 (0.22)
	White shrimp	Pre-spill	16.81 (1.63)	9.73 (0.06)
		2011	63.10 (24.11)	10.63 (0.27)
		2012	20.42 (3.28)	10.84 (0.21)
Lake Calcasieu	Brown shrimp	Pre-spill	102.57 (4.16)	4.44 (0.05)
		2010	109.18 (18.85)	4.01 (0.31)
		2011	126.18 (22.63)	4.09 (0.27)
		2012	89.12 (21.63)	4.19 (0.31)
	White shrimp	Pre-spill	37.89 (1.67)	7.48 (0.05)
		2011	17.52 (3.33)	8.97 (0.37)
		2012	12.27 (2.45)	8.97 (0.33)
L. Pontchartrain/Breton Sound	Brown shrimp	Pre-spill	45.75 (2.29)	6.88 (0.05)
		2010	38.22 (6.76)	7.47 (0.22)
		2011	88.94 (16.92)	5.68 (0.43)
		2012	94.65 (26.80)	4.95 (0.42)
	White shrimp	Pre-spill	26.05 (1.49)	8.44 (0.05)
		2011	34.35 (5.32)	8.80 (0.24)
		2012	9.21 (1.73)	11.04 (0.46)
Terrebonne Bay	Brown shrimp	Pre-spill	51.78 (1.63)	6.01 (0.03)
		2010	45.90 (6.32)	5.38 (0.23)
		2011	118.64 (10.63)	4.50 (0.21)
		2012	104.85 (9.65)	4.22 (0.20)
	White shrimp	Pre-spill	30.91 (1.21)	8.83 (0.04)
		2011	69.29 (9.71)	8.60 (0.22)
		2012	33.85 (4.49)	9.66 (0.23)
Vermillion Bay/Teche Basin	Brown shrimp	Pre-spill	52.35 (1.94)	5.13 (0.04)
		2010	126.37 (21.52)	4.47 (0.22)
		2011	56.75 (6.09)	4.89 (0.17)
		2012	70.85 (10.18)	3.78 (0.21)
	White shrimp	Pre-spill	66.53 (1.84)	7.20 (0.05)
		2011	62.20 (6.28)	8.09 (0.24)
		2012	60.15 (6.03)	7.80 (0.26)

Parentheses include standard error.

**Figure 3:**
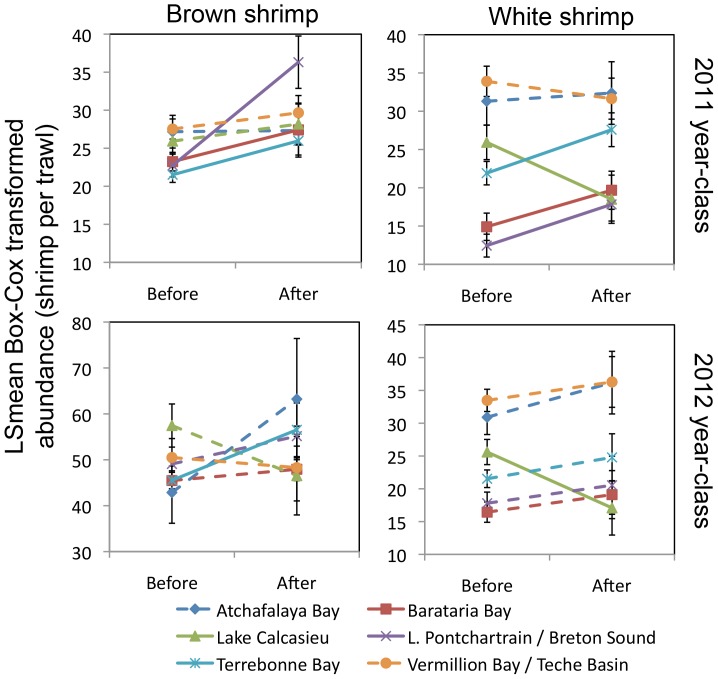
Abundance of Gulf shrimp post-spill as compared to pre-spill for 2011 and 2012 year-classes. Solid lines indicate a significant difference between before and after abundances, while dashed lines indicate insignificant effects.

**Figure 4:**
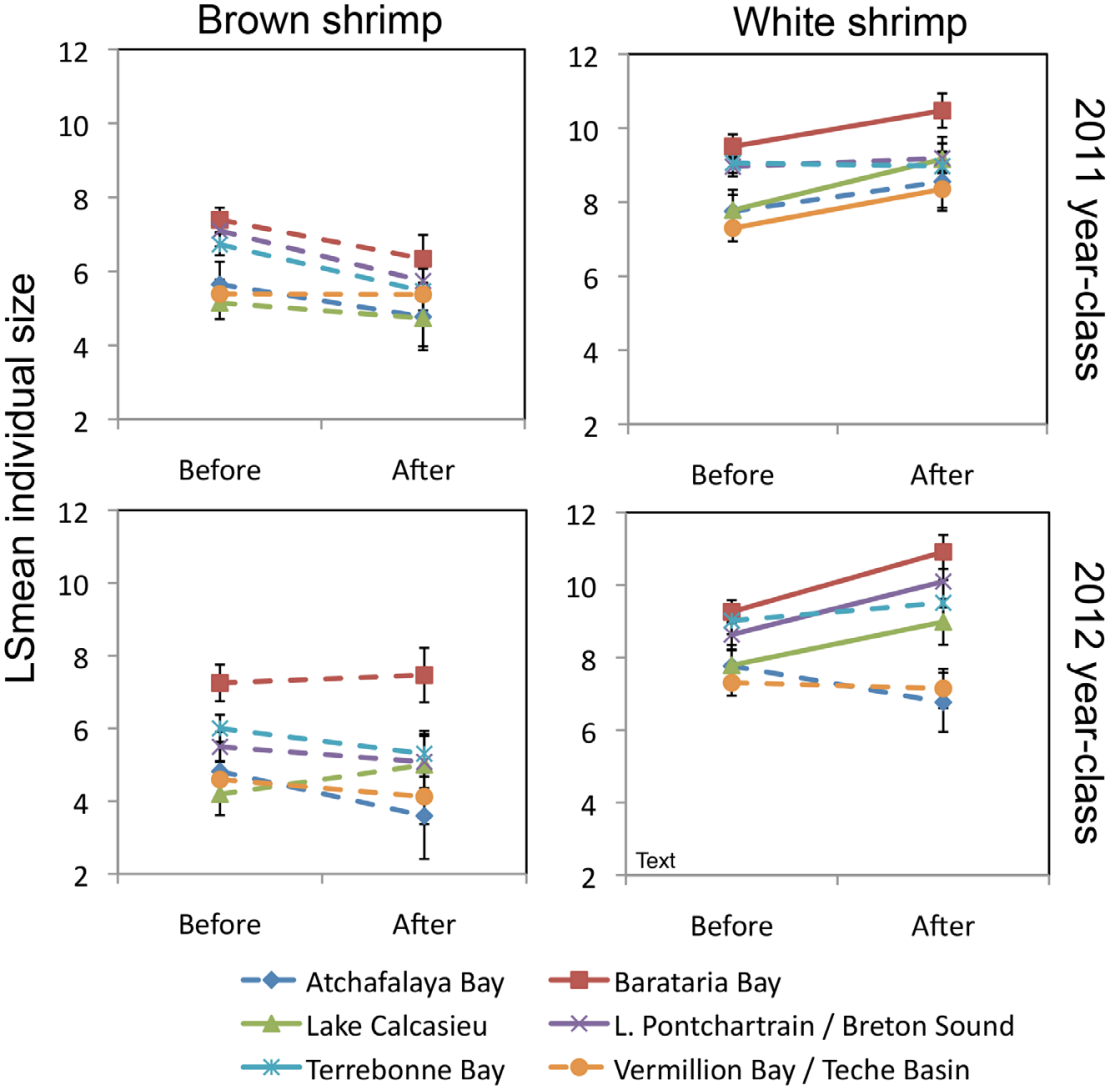
Size of Gulf shrimp post-spill as compared to pre-spill for 2011 and 2012 year-classes. Solid lines indicate a significant difference between before and after abundances, while dashed lines indicate insignificant effects.

In the large-scale analysis, the LSmean contrast tests of the 2010 brown shrimp year-class showed no significant changes in either abundance or size, in any of the basins compared with before the spill. In the 2011 year-classes however, both brown and white shrimp abundances were higher than pre-spill abundances in basins that were affected by the spill (both species: Barataria Bay, Lake Pontchartrain/Breton Sound, and Terrebonne Bay) whereas this effect on abundance was not seen in basins that were minimally affected (both species: Atchafalaya Bay and Vermillion Bay/Teche Basin) (Table 3; Figure 3). In Lake Calcasieu, which we consider not affected by the spill, brown shrimp were equally abundant after the spill as before, but white shrimp were less abundant after the spill than before the spill (Table 3; Figure 3). The pattern of increased abundance of post-spill shrimp in affected basins seen in 2011 year-classes was absent in 2012 year-classes, except for brown shrimp in Terrebonne Bay (Table 3; Figure 3). White shrimp in Lake Calcasieu showed the same post-spill decrease in abundance as in the 2011 year-class (Table 3; Figure 3). Size of brown shrimp in any of the basins did not significantly differ after the spill compared with before the spill, in either the 2010, 2011, or 2012 year-classes. The size of post-spill white shrimp was significantly larger in both affected and minimally affected basins, in both 2011 and 2012 (Table 3; Figure 4).

**Table 3:** Significant before-after (spill) differences in abundance and size of both brown and white shrimp in each basin.

Abundance - 2011 year-class		contrast estimate (SE)	F ratio	*p*
Brown shrimp	Barataria Bay	8.3(3.6)	5.4	0.02
	L. Pontchartrain/Breton Sound	27.2 (6.4)	18.0	<0.01
	Terrebonne Bay	8.9 (3.2)	7.2	<0.01
White shrimp	Barataria Bay	9.4 (3.9)	5.8	0.02
	Lake Calcasieu	−14.8 (4.6)	10.5	<0.01
	L. Pontchartrain/Breton Sound	10.7 (3.6)	9.0	<0.01
	Terrebonne Bay	11.3 (3.4)	11.0	<0.01
**Abundance - 2012 year-class**				
Brown shrimp	Terrebonne Bay	22.0 (10.6)	4.3	0.05
White shrimp	Lake Calcasieu	−17.0 (7.6)	5.0	0.03
**Size - 2011 year-class**				
Brown shrimp	Terrebonne Bay	−2.5 (1.2)	1.2	0.05
White shrimp	Barataria Bay	1.9 (0.7)	6.5	0.01
	Lake Calcasieu	2.7 (0.9)	9.2	<0.01
	Vermillion Bay/Teche Basin	2.1 (0.7)	8.3	<0.01
**Size - 2012 year-class**				
White shrimp	Barataria Bay	3.2 (0.8)	15.3	<0.01
	Lake Calcasieu	2.4 (1.0)	5.7	0.02
	L. Pontchartrain/Breton Sound	2.9 (1.3)	4.9	0.02

Before data (1987–2010) are compared with one year after the spill (2011 year-class) and two years after the spill (2012 year-class).

## Discussion

A consistent pattern emerges from our two analyses: the abundance of both brown and white shrimp was significantly higher after the spill occurred. Even though this trend is clearly present for both shrimp species in the 2011 year-class, it is absent in nearly all basins in the 2012 year-class. We found inconsistent results regarding mean size, where the small-scale analysis suggested a reduction in size of white shrimp, while the large-scale analysis suggested an increase in size of post-spill white shrimp. In addition, the increase in size found in the large-scale analysis occurred in basins that were impacted by the spill (Barataria Bay and Lake Pontchartrain/Breton Sound) as well as in basins that were minimally impacted by the spill (Lake Calcasieu and Vermillion Bay/Teche Basin), indicating that this was not an effect of the spill. The lack of consistency in the findings regarding the size for both species, in either analysis, may indicate that the mean size of shrimp was unchanged by the occurrence of the spill.

We pose two hypotheses that explain the increase in abundance; one suggests that the increase in abundance is a result of a direct effect of the spill on shrimp, whereas the other suggests it to be an effect of fisheries management as response to the spill. First, as the spilled oil entered coastal estuaries, it introduced various pollutants, including PAHs [3]. The growth rate reducing effect of PAHs [11]–[14] can have an effect on the emigration behavior of shrimp. For both brown and white shrimp, individual size is a determining factor in emigration behavior; individuals will migrate from the estuaries to deeper offshore waters at a threshold size [10]. Rozas et al. [25] observed brown shrimp kept in mesocosms on oiled marsh edge to exhibit a decreased individual growth rate directly after the spill. Such decreased growth rates may have led to delayed offshore migration and resulted in increased abundances inshore. This retention of sub-adults inshore would also result in inconsistent and non-significant changes in mean size of shrimp, even though individual growth rates are reduced as shown by Rozas et al [25].

Second, the increase in inshore shrimp abundance may have been caused by the decrease in fishing pressure in the affected estuaries. In response to the spill, the LDWF closed affected estuaries to the harvest of shrimp. Recreational and commercial fishing closures for the majority of Barataria Bay and Terrebonne Bay went into effect during May 2010 and lasted approximately 3 to 4 months. Fishing closures in or near Atchafalaya Bay, Lake Pontchartrain/Breton Sound, and Vermillion Bay/Teche Basin were limited to outside waters, and were of short duration. No fishing closures were established in or near Lake Calcasieu (wlf.louisiana.gov/category/news/oil-spill-closures; data not shown). The duration of closures depended on the severity of the spill impact, and arguably as a result, the shrimp abundance increased most in the heavily impacted estuaries due to the decreased efforts of the shrimp fisheries. In concordance with this theory, the abundance of white shrimp decreased in Lake Calcasieu, which is the Louisiana basin furthest away from the spill; this could have been a result of the increased fishing pressure in basins that suffered the lowest spill impact. Since both events happened simultaneously and proportionally (more heavily impacted sites had a higher reduction in fishing pressure), the relative contribution of the events on the abundance of shrimp cannot be separated in our analyses. Potentially, both events contributed to our finding.

The trend of increased abundances was less prevalent in the 2012 year-classes. This may indicate that the increased inshore abundance of shrimp was a short-lived effect. The rebound to normal abundance and the absence of any effect on shrimp size agrees with the view that the spill may have negligible long-term effects on Louisiana shrimp [20]. However, long-term effects of the spill on shrimp may manifest in other traits, such as compromised immunological or life-history traits. Studies that focus on physiological responses of shrimp to harmful compounds in oil such as PAHs would help determine other sub-lethal effects, and distinguish oil spill effects from co-occurring events, such as fishing closures.
